# Flash Characterization of Smartphones Used in Point-of-Care Diagnostics

**DOI:** 10.3390/bios12121060

**Published:** 2022-11-22

**Authors:** Binh V. Vu, Rongwei Lei, Chandra Mohan, Katerina Kourentzi, Richard C. Willson

**Affiliations:** 1 William A. Brookshire Department of Chemical and Biomolecular Engineering, University of Houston, Houston, TX 77204, USA; 2 Department of Biomedical Engineering, University of Houston, Houston, TX 77204, USA; 3 Department of Biology and Biochemistry, University of Houston, Houston, TX 77204, USA; 4 Escuela de Medicina y Ciencias de la Salud ITESM, Monterrey 64710, NL, Mexico

**Keywords:** point of care, diagnostics, smartphone, flash, iPhone, battery, variation, spectrum, intensity

## Abstract

Rapidly growing interest in smartphone cameras as the basis of point-of-need diagnostic and bioanalytical technologies increases the importance of quantitative characterization of phone optical performance under real-world operating conditions. In the context of our development of lateral-flow immunoassays based on phosphorescent nanoparticles, we have developed a suite of tools for characterizing the temporal and spectral profiles of smartphone torch and flash emissions, and their dependence on phone power state. In this work, these tools are described and documented to make them easily available to others, and demonstrated by application to characterization of Apple iPhone 5s, iPhone 6s, iPhone 8, iPhone XR, and Samsung Note8 flash performance as a function of time and wavelength, at a variety of power settings. Flash and torch intensity and duration vary with phone state and among phone models. Flash has high variability when the battery charge is below 10%, thus, smartphone-based Point-of-Care (POC) tests should only be performed at a battery level of at least 15%. Some output variations could substantially affect the results of assays that rely on the smartphone flash.

## 1. Introduction

Smartphones are approaching ubiquity in most areas of the world, and have naturally emerged as an important basis of diagnostic and assay technologies for point-of-need and low-resource settings applications [1,2,3,4]. Due to their powerful internal computers, optical sensors, light sources, global positioning systems (GPS), and connectivity, smartphones are nearly ideal readout systems [5]. While an impressive variety of smartphone functions have been adapted to these applications, perhaps the majority of interest has focused on the photographic functions of smartphones, especially their digital cameras and solid-state flash and illumination functions [6,7,8,9,10,11,12].

There are many types of smartphone-based assays, using a variety of formats and reporters. Particularly prominent are lateral-flow and vertical-flow immunoassays, which have been applied to analytes ranging from pathogens, DNA amplicons, and vitamins, to host biomarkers of disease [13]. Optical readouts of these assays include established techniques such as imaging of fluorophores and light-absorbing gold and colored latex nanoparticles, as well as newer techniques such as microbubbling assay [7,14,15,16,17,18,19,20,21,22], and our own recently introduced phosphorescent nanoparticles [23,24,25,26]. Smartphones have gradually replaced desktop scanners and emerged as suitable low-cost readers for microfluidic devices, specifically paper-based colorimetric immunoassays [27]. When imaging a colorimetric test, these smartphone-based assays require a light source, usually the phone’s own flash, to avoid variability due to changes in the ambient lighting [5,7]. Of special relevance is the development of an optical platform for the imaging of urinalysis reagent strips which provides for diffusion of the light from the flash to avoid any artifacts caused by low or variable ambient light and ensure homogenous illumination of the strips [28]. Variations in ambient lighting were found potentially to significantly compromise the robustness of smartphone-based gold nanoparticle LFAs [29]. Consequently, most smartphone-based assay designers use the phone’s flash to illuminate or excite the reporters, and assume that the flash produces consistent light output.

The smartphone-based nanophosphor assays developed by our group and by Luminostics, Inc. (now Clip Health) use the internal smartphone flash to excite the reporters. While their excellent signal-to-noise ratios and simple excitation and detection modes make nanophosphors very attractive as lateral flow reporters, their energy-storage mechanism, their wavelength-dependent excitation, and the rapid decay of their emission intensity make nanophosphor assays dependent upon the flash performance of the smartphone used to excite and image them. To support smartphone-based nanophosphor lateral flow assays (LFAs) we have developed a suite of tools for characterizing smartphone flash performance and uniformity. These tools are described and made easily available here, and some conclusions about smartphone flash performance are related.

## 2. Materials and Methods

**Figure 1:**
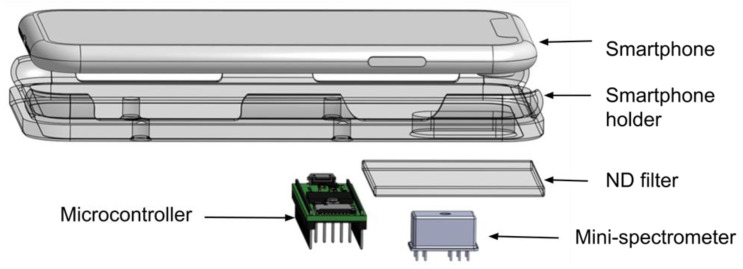
Exploded view of the smartphone flash spectrometer. The device is comprised of a smartphone adapter, neutral density filters (total optical density 4 OD, Newport), a micro-spectrometer C12880MA (Hamamatsu), a microcontroller (Teensy 3.6, 32 bit 180 MHz ARM Cortex-M4 processor, PJRC), and a microSD card, and is packed into a 3D-printed enclosure (https://github.com/willsonlab/mini-spectrometer, accessed on 1 September 2022).

We developed a fast spectrometer intended for phone characterization, and its accompanying firmware and software. The system can acquire more than 150 spectra (340–850 nm) per second with a spectral resolution of 11.8 nm, a minimum integration time of 1 ms, and a read delay of 5 ms. The firmware was modified from that of Versek et al., which was used to test various alcoholic beverages and oils [30]. We added the ability to read multiple spectra at high speed, store them on an SD card and transfer data to Excel VBA. The spectrometer system is composed of a micro-spectrometer C12880MA (Hamamatsu Photonics, Hamamatsu City, Shizuoka Pref., Japan), and a microcontroller (Teensy 3.6, 32-bit 180 MHz ARM Cortex-M4 processor, PJRC.COM, LLC., Sherwood, OR, USA), neutral density filters (total optical density 4 OD, Newport, Irvine, CA, USA), a microSD card, a 3D-printed enclosure, and smartphone adapters. (Figure 1). The C12880MA is a pre-calibrated, high-sensitivity, ultra-compact (finger-tip sized, 20.1 × 12.5 × 10.1 mm), hermetically sealed spectrometer head. It has 288 pixels with a typical and maximum spectral resolution of 12 nm and 15 nm, respectively. The spectrometer wavelength accuracy of the complete system was validated with a mercury argon calibration light source (HG-1, Ocean Optics, Orlando, FL, USA). The whole system cost less than $300, with the mini-spectrometer constituting the majority ($250). The Teensy 3.6 microcontroller was chosen for its high speed, 16-bit ADC, and built-in micro SD card slot. The device was connected to a computer by a USB cable with serial communication.

Data acquisition and processing were done with VBA macros using the modCOMM VBA module written by David M. Hitchner [31] and transferred to an Excel spreadsheet. Four different models from two major smartphone brands (Apple, Cupertino, CA, USA and Samsung, Seoul, South Korea) were measured, including Apple iPhone 6s, iPhone 8, iPhone XR and Samsung Note 8. A custom smartphone holder for each model was 3D-printed with black PLA filament (Flashforge Pro, Zhejiang Flashforge 3d Technology Co., Ltd., Jinhua, China). All codes and 3D coordinate models are available at our GitHub repository [32]. Flash measurements were performed on phones at various battery states of charge (from 2% to 100%), with and without being plugged into the charger. The flashes were activated by a smartphone native camera app in manual mode with flash set to “On” and manual focus (for Samsung, Seoul, South Korea) or AE/AF Lock (for Apple, Cupertino, CA, USA). Newer smartphone models (iPhone 8, XR, and Note 8) employ pre-exposure light measurement as feedback to adjust the flash and exposure conditions. Measurements were taken with the camera and sensor covered with black aluminum tape (T205-1.0, Thorlabs, Newton, NJ, United States) while only the flash light was allowed to pass through a circular cutout. Flash activation was done manually by successively touching the capture button at the rate of 2 taps/second for 10 s and the data acquisition was recorded for 10 s. Since the finger presses were more frequent than the maximum flash rate, the timings between successive flashes/captures were automatically determined by the smartphone. For the iPhone 6s, the rate was ca. 6 flashes/captures in 10 s. The reported coefficients of variation (CV) were determined at the wavelength with the highest variation.

A model lateral flow assay for the detection of Cystatin C was constructed in-house with Whatman FF120HP nitrocellulose membrane (Cytiva, Marlborough, MA, USA) assembled on an adhesive backing card (MIBA-020; DCN Diagnostics, Carlsbad, CA, USA) together with Whatman Standard 14 sample pad and Whatman CF 5 absorbent pad (Cytiva, Marlborough, MA, USA). The LFA cards were striped with 0.72 mg mL^−1^ mouse monoclonal anti-cystatin C antibodies (DY1196, R&D Systems, Minneapolis, MN, USA) for the test line and 1 mg mL^−1^ goat polyclonal anti-mouse antibodies (ABGAM-0500; Arista Biologicals, Allentown, PA, USA) for the control line at a rate of 1 mL cm^−1^ using a BioDot dispenser (XYZ30600124, BioDot, Irvine, CA, USA). The striped membrane was dried at 37C for 2 h in an incubator (Micro Hybridization Incubator 2000, Robbins Scientific, San Diego, CA, USA) and then cut into 3 mm wide strips using a ZQ2000 Guillotine Cutter and stored in a desiccator until used.

Silica-encapsulated and silanized SrAl2O4:Eu^2+^,Dy^3+^ (SAO) nanophosphors were prepared as we previously described in Danthanarayana et al. [26]. 2 mg SAO nanophosphors were suspended in 700 µL of PBS, pH 8 containing 50 mg of rabbit polyclonal anti-cystatin c antibodies (A1561, ABclonal Science, Inc, Woburn, MA, USA) and 250 µL of 1 M NaBH_3_CN (Chem-Impex International, Inc., Wood Dale, IL, USA) in PBS, pH 8. The mixture was sonicated for 5 min and placed on a rotator at 20 rpm for 2 h at room temperature. Finally, the SAO nanophosphor reporters were washed with PBS, pH 7.4, and then passivated with 40 mg mL^−1^ bovine serum albumin (BSA; 98%; Sigma-Aldrich, St. Louis, MO, USA). The functionalized anti-cystatin c SAO reporters were stored in 100 µL of borate storage buffer (10 mM sodium borate (J.T. Baker, Phillipsburg, New Jersey, USA), 150 mM NaCl (Macron, Radnor, PA, USA), 0.1% BSA, 0.04% PVP-40 (Sigma-Aldrich, St. Louis, MO, USA), 0.025% Tween-20 (Sigma-Aldrich, St. Louis, MO, USA), pH 8.5) at 4C.

Recombinant cystatin C standard protein (component of the ELISA DuoSet DY1196, R&D Systems, Minneapolis, MN, USA) was spiked into LFA running buffer (10 mM HEPES, 0.6% PVP40, 0.4% PEG, 100 mM NaCl, 1% BSA, pH 7.25) at 750, 500, 250, 125, 62.5 and 31.25 ng mL^−1^. Forty µL of protein solution (or LFA running buffer for the no-analyte negative control) with 10 µg of SAO reporters (0.5 µL) were added onto the sample pad of the LFA strips and were allowed to run for 40 min. An iPhone 5s equipped with a 3-D printed attachment was used for imaging. A proprietary software application called ‘Luminostics’ was used to control the iPhone’s flash and rear camera. The app turned on the flash for 3 s to excite the nanophosphors, switched off the flash, then captured the images with the iPhone built-in camera after a 100 ms time delay. The camera settings used were ISO 2000 and 0.5 s exposure time. The camera captures four images, averages the images and gives the average result. Imaging of the LFA strips was done at three battery levels: 100% with charger plugged in, 15–25% unplugged, and below 10% unplugged. For brevity, we call the three battery levels 100%, 20%, and 10%. Each analyte concentration was imaged three times. ImageJ [33] was used to extract the intensity profiles from the images and the ratio of the integrated intensities of the test line (TL) and control line (CL) was calculated for each analyte concentration.

## 3. Results and Discussion

Smartphone flash operation demands significant power over a very short time. The charge level of the smartphone battery can affect flash variability [25], duration, intensity, and spectral characteristics.

### 3.1. Flash Duration and Variability as a Function of Power State

The typical flash duration is around 100 ms. The traditional camera flash uses a flashtube filled with inert gas and a high-voltage circuit to ionize the gas and generate an arc discharge. Due to size constraints and voltage requirements, with the exception of the Nokia Lumia 1020, smartphones use a phosphor-based white LED as flash. Most white LEDs are based on a blue Indium Gallium Nitride (InGaN) LED coated with phosphors of different colors to produce white light [34].

**Figure 2:**
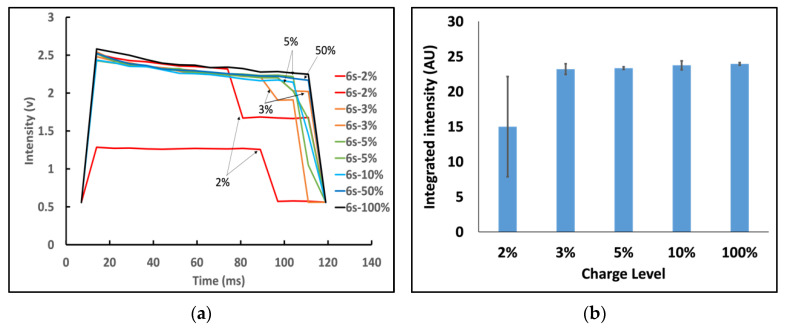
Effects of battery level on flash intensity and duration for unplugged iPhone 6s. (**a**) Variations of flash intensity at different charge levels. (**b**) Integrated flash intensity over the duration of flash. At high charge (>5%), flash duration and intensity were very consistent. However, at low charge (<5%), the flash intensity decreased and the duration became shorter. Flash intensities were measured at 437 nm, the highest peak in the flash LED emission spectrum (n = 3).

To test the impact of battery charge level, flash duration measurements were performed at various states of charge battery (from 2% to 100%), with and without being plugged into the charger. For ease of comparison, the flash intensities were compared at 437 nm, the highest peak in the flash LED emission spectrum. At high charge, the flash duration was very consistent. However, we found that at low charge (<5%), the flash intensity and duration varied significantly, most prominently with the iPhone 6s (Figure 2). At the extreme low state of charge (<2%), the intensity was less than half of the normal level. Below 5% charge, the smartphone could not sustain the normal flash intensity for the duration of the flash period and dipped down at the end. This could substantially affect the results of smartphone-based biomedical assays since the total excitation energy was reduced. For example, our phosphorescent nanoparticles are charged by the flash energy over the entire flash duration [23]. Significant variation in flash intensity or duration could significantly affect subsequent light emission, and a typical intensity check would not detect this shortfall.

**Figure 3:**
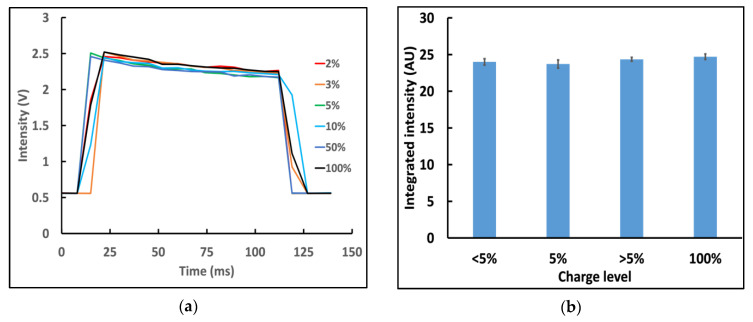
Plugging into the charger makes the iPhone 6s flash more consistent. (**a**) Variations of flash intensity (437 nm) at different charge levels while plugged into the charger. (**b**) Integrated flash intensity over the duration of the flash. When plugged in, the iPhone 6s had consistent flash output regardless of battery charge (n = 3).

For some smartphones (e.g., iPhone 6s) plugging in the charger restores flash performance to a normal level regardless of the state of charge (Figure 3).

**Figure 4:**
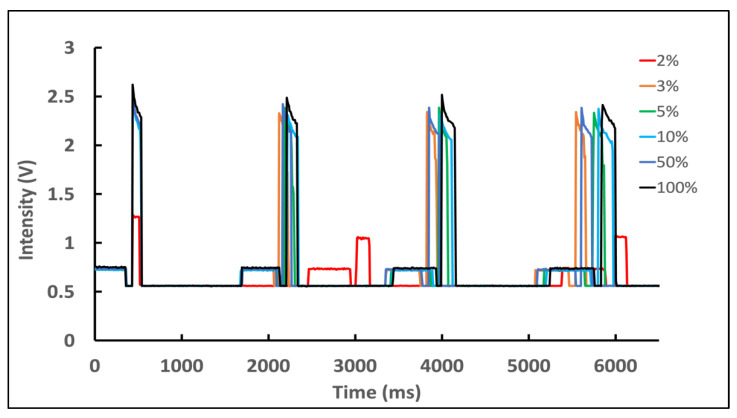
Elongated recovery time with iPhone 6s at extreme low state of charge. 437 nm flash intensity at 2% (red) was significantly reduced and the phone took a long time to recover for subsequent flash activation. The smartphone was rested at the start of each measurement series.

At normal charge, the flash/capture rate and intensity level were very consistent. At a very low charge (<2%), the lag times between the flashes were elongated and the intensities of the later flashes were reduced (Figure 4). These measurements were collected starting with a phone rested for at least 5 min, and with only a native camera app running to eliminate any delay caused by other applications. Similar results were also found with the iPhone 5s (Figure S1).

### 3.2. Spectral Variation as a Function of Power State

**Figure 5:**
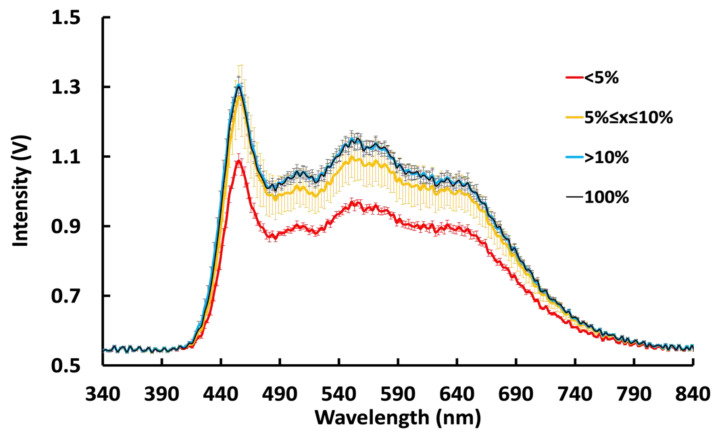
Variation of unplugged iPhone XR flash emission spectra with battery charge level. Below 5% (red), the flash output was consistently below the nominal level. At battery levels between 5% and 10%, the flash output varied widely and unpredictably with the max CV and average CV of 8.2% and 2.7%, respectively. At greater than 10% battery level, the flash output was consistently at a nominal level with the max CV of 5.5% and average CV of 1.1%. Measurements were taken twice at each battery charge level on three iPhone XR’s.

Most smartphone flash-based assays are particularly sensitive to a certain spectral range of the flash output. Any variations in the flash spectral distribution could greatly affect the assay results, especially with fluorescence-based assays. To test for spectral variation, the emission spectra of smartphone camera flashes were measured at various battery charge levels. As shown in Figure 5, at the extreme low state of charge (less than 5% for iPhone 6s and 2% for iPhone XR), the flash intensity was significantly and consistently lower than the nominal output. At charge between 5% and 10%, the intensity of the flash output had a very wide variation (max CV and average CV of flash output for three iPhone XRs in duplicate readings was 8.2% and 2.7%, respectively and was unpredictable. Above 10%, the variation of the flash output was narrower (max CV of 5.5% and average CV of 1.1%). Unlike the previous model, 6s, plugging in the iPhone XR did not affect the flash output (SI2). This suggests there were changes in the circuitry of the newer iPhone models making them powered exclusively by the battery.

**Figure 6:**
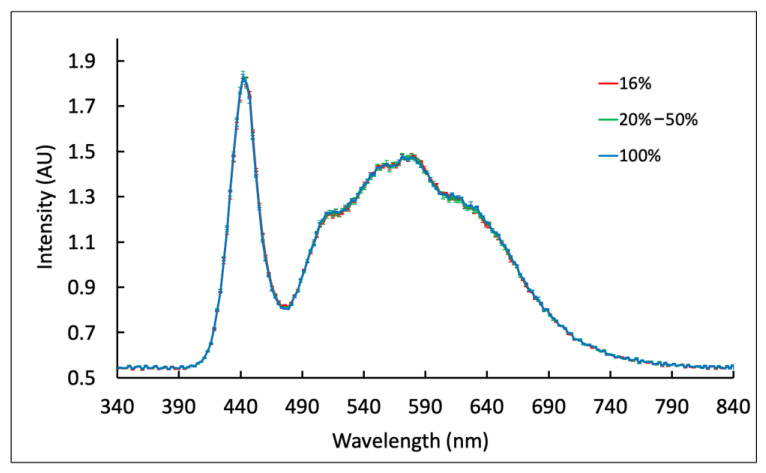
Flash spectrum variation of unplugged Samsung Note8 at various battery charge levels. The flash output was very consistent at various battery charge levels from 16% to 100%, with an average CV of 1.6% ± 0.4. Flash performance for battery levels below 16% was not measured due to Samsung software restrictions. Measurements were taken three times at each battery charge level on two Samsung Note 8′s.

Testing of Samsung Note 8 flash variability at low charge was not possible because of a software restriction that prevents flash usage at charge below 15%, even when plugged in. The performance of the flash was very consistent from 16% to 100% charge when unplugged or plugged in, with an average intensity CV of 1.6% ± 0.4 (Figure 6).

### 3.3. Intra-Model Flash Variation

**Figure 7:**
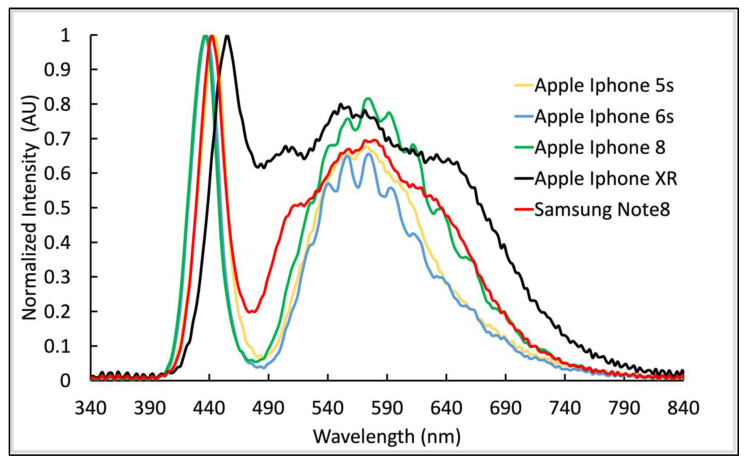
Normalized emission spectra of tested smartphones. Flash spectra of Apple iPhone 5s (yellow), iPhone 6s (blue), iPhone 8 (green), iPhone XR (black), and Samsung Note 8 (red) were normalized to their respective highest values for ease of comparison since each model required a custom 3D-printed holder.

Among the different phones of the same model tested, the flash output at higher charge was fairly consistent (CV of 3.4% ± 0.9 for Note 8; n = 2 and 4.5% ± 0.9; n = 3 for iPhone XR), for a limited number of samples. The spectral distribution of the flashes was very consistent within a smartphone model, despite high variability in flash intensity at lower charge. The flashes of most smartphones share similar spectral characteristics, with a high, narrow peak at around 435 nm to 455 nm and a broader peak from 500 nm to 700 nm (Figure 7). For most smartphones, there is much lower light intensity between the two main peaks, around 480 nm. This spectral gap limits sensitivity for reporter molecules or particles that are excited or absorb at 460 nm to 510 nm (e.g., fluorescein). One exception is the Apple iPhone XR’s Quad-LED True Tone flash used for color tone correction, which significantly reduces this spectral gap (Figure 7).

**Figure 8:**
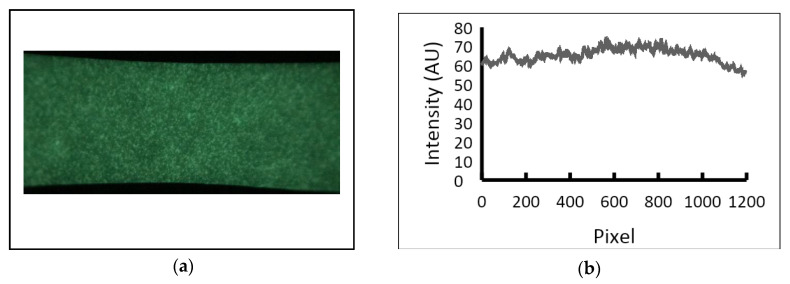
Spatial variability of camera sensitivity. (**a**) Image of a uniformly phosphorescent strip excited with an external light source and captured with our LFA reader attachment. (**b**) The image was analyzed with ImageJ to create an intensity profile.

We also evaluated the spatial variability of camera sensitivity using a control LFA cartridge containing a uniformly phosphorescent film (FDC 2847 RTape GlowEFX). The control cartridge was pre-flashed with a diffuse light source and imaged in our attachment with an iPhone 8 Plus. The image was then analyzed using ImageJ [33]. The resulting intensity profile showed that the sensitivity in the middle field of view is about 10% greater than at the outside edges (Figure 8). This is due to the pincushion distortion introduced by the lens in our attachment.

### 3.4. Effect of Battery Level on Nanophosphor Reporter-Based LFA Results

**Figure 9:**
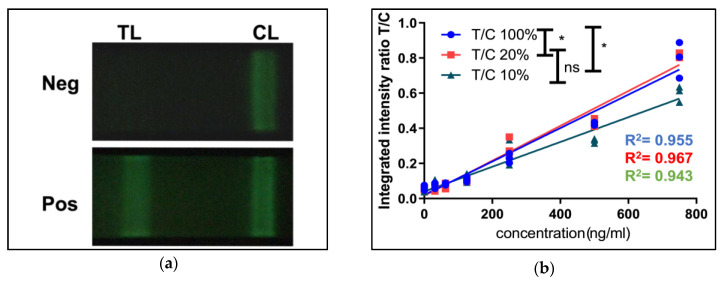
Effect of battery charge level on the performance of nanophosphor-based LFA. (**a**) Typical images of negative (top; no-analyte) and positive (bottom; 500 ng mL^−1^) nanophosphor-based LFA strips. Nanophosphor-based LFA strips were excited by the flash on an iPhone 5s smartphone and the images were immediately captured using the built-in camera to capture the emitted light from the test line (TL) and control line (CL) as described in Methods. (**b**) T/C ratios of cystatin C titration series imaged with an iPhone 5s and the LFA attachment under 3 battery charge levels (<10%, 15–25%, and 100%). *, *p* < 0.05, ns, *p* > 0.05 as determined using paired *t*-test.

We tested the impact of the battery charge level on the performance of a nanophosphor-based LFA to measure cystatin C, a biomarker of renal function [35,36,37,38]. Developed in our lab, this nanophosphor-based LFA test uses the flash on an iPhone 5s smartphone to excite the nanophosphor reporters captured on the test and control lines and the built-in camera to capture the emitted light from the test line (TL) and control line (CL) (Figure 9a). The ratio of TL over CL (T/C) of an LFA test (instead of only the signal at the test line) is typically used as the final readout to compensate for flow inconsistencies. We used a cystatin C standard curve to evaluate the flash performance of the iPhone 5s at various battery charge levels. The results showed that the flash performance (as assessed by the TL/CL ratio) was significantly impacted when the battery charge level was below 10%. At 100% plugged-in and 20% unplugged (actual level: 15% to 25%) states, the T/C values were overall higher than those in 1-10% unplugged state (*p* < 0.05) (Figure 9b). This indicates the low battery level can result in poorer linearity of the standard curve and generally lower T/C. So, the lower total excitation energy delivered to the reporters by the flash at low battery charge could significantly affect the performance of smartphone-based biomedical assays.

## 4. Conclusions

Most lab-based diagnostic instruments incorporate means for routine calibration tests to ensure a high level of accuracy and precision and are typically used with internal controls and calibrators. In smartphone-based POC tests, camera and flash variability are often not investigated in detail, and the small size of smartphone-based POC tests hinders the implementation of internal controls and calibrators. We engineered a new, fast spectrometer and accompanying firmware and software intended for smartphone flash characterization and made these publicly available to enable implementation by researchers and test developers. Using these tools, we established protocols and design parameters to minimize measurement variability when using smartphones to read LFAs and other types of tests. We documented that the flash has high variability when the state of charge of the battery is below 10%, thus, smartphone-based POC tests should only be performed with a minimum battery level of at least 15% to provide enough safety margin. Some smartphone manufacturers have started to implement camera flash restriction when battery level is below 15%, but this is not universally implemented yet. Nevertheless, this requirement can be programmed into the smartphone app, to block the measurement function when the battery is below 15%. When the battery charge is more than 15%, flash has very small variation (average CV of 2.2% ± 0.6 for iPhone XR or 1.6% ± 0.4 for Note 8). As smartphone cameras become more sophisticated, camera flashes are also becoming more complicated, with multiple LEDs packaged in a flash, light sensors, and complex flash control software for color tone correction. Developers of smartphone-based POC tests must be able to fully understand, and ideally control, the duration, intensity, and the color characteristics of the flash to minimize signal variation and take full advantage of the growing capabilities of modern smartphones.

## Data Availability

The data presented in this study are openly available in https://github.com/willsonlab (accessed on 15 August 2022).

## Supplementary Materials

The following are available online at https://www.mdpi.com/article/10.3390/bios12121060/s1, Figure S1: Effects of battery level on flash intensity and duration for unplugged iPhone 5s; Figure S2: Variation of plugged in iPhone XR flash emission spectra with battery charge level; Code S1: Firmware for Teensy microcontroller to interacting with the Hamamatsu C12880MA.
