# Supplementary material for: Flash Characterization of Smartphones Used in Point-of-Care Diagnostics
Source: Biosensors (Basel). 2022 Nov 22;12(12):1060. doi: 10.3390/bios12121060 (PMC9776052; doi:10.3390/bios12121060)
Supplement: Supplementary file 1 [file biosensors-12-01060-s001.zip › biosensors-1941953-supplementary.pdf]

biosensors

MDPI

Supplementary

Flash Characterization of Smartphones Used in Point-of-Care Diagnostics

Figure S1. Variation of plugged-in iPhone XR flash spectrum with battery charge level.

Code S1. Firmware for Teensy microcontroller to interact with the Hamamatsu C12880MA

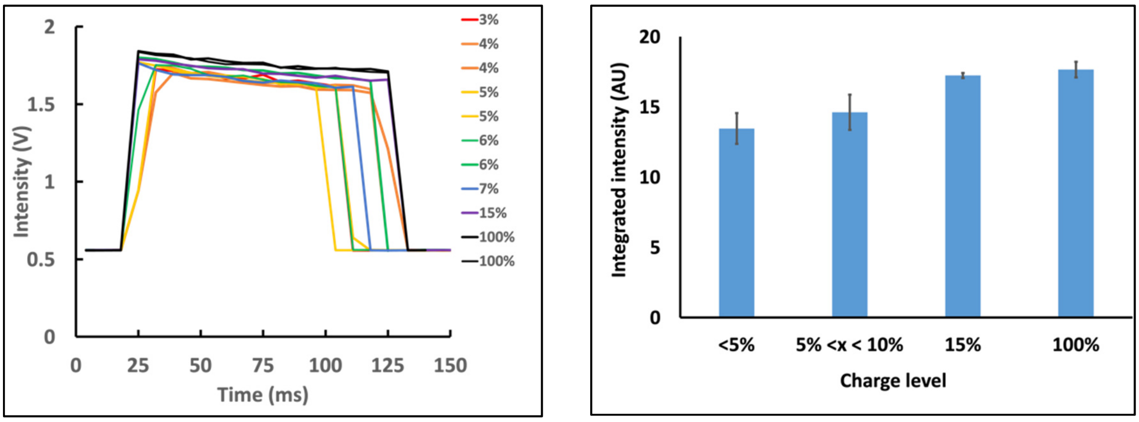

(a) (b)

Figure S1. Effects of battery level on flash intensity and duration for unplugged iPhone 5s**.** (a) Variations of flash intensity at different charge levels. (b) Integrated flash intensity over the duration of flash. At high charge (>15%), flash duration and intensity were very consistent. However, at low charge (<10%), the flash intensity decreased and the duration became shorter. Flash intensities were measured at 437 nm, the highest peak in the flash LED emission spectrum (n=3).

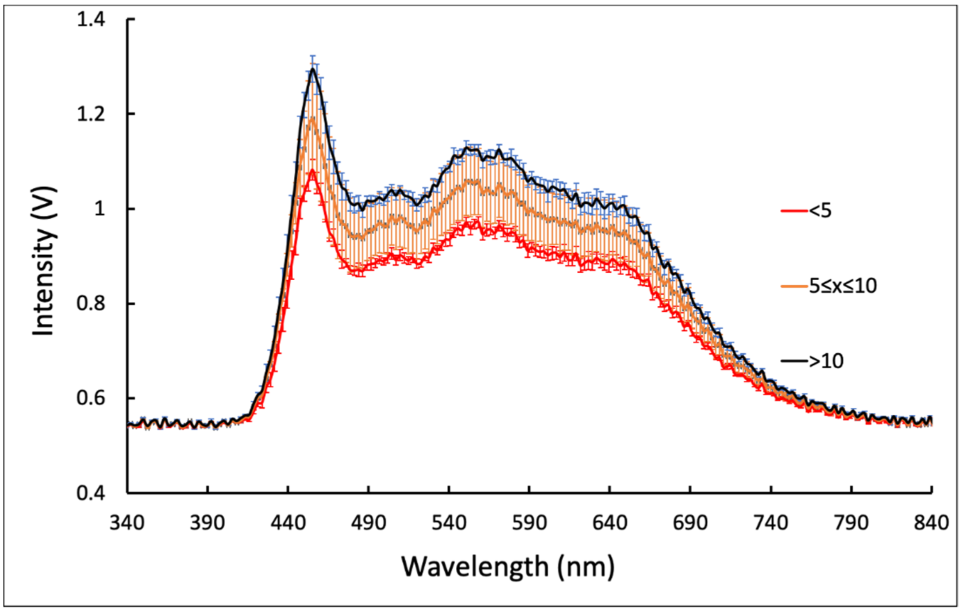

Figure S2. Variation of plugged-in iPhone XR flash spectrum with battery charge level. Below 5% (red), the flash output was consistently below the nominal level. At battery levels between 5% and 10%, the flash output varied widely and unpredictably with the max CV and average CV of 10.4% and 3.0%, respectively. At greater than 10% battery level, the flash output was consistently at a nominal level with max CV of 3.8% and average CV of 1.0%. Measurements were taken twice at each battery charge level and plugged in on three iPhone XR's.

Code S1. Firmware for Teensy microcontroller to interacting with the Hamamatsu C12880MA.

```
/******************************************************************************
Firmware for Teensy to interact with the Hamamatsu C12880 microspectrometer,
  which uses the c12880.h library
******************************************************************************/

#include <elapsedMillis.h>
#include "c12880.h"
#include <SD.h>
#include <Arduino.h>

#define SPEC_TRG                A0
#define SPEC_ST                 A1
#define SPEC_CLK                10
#define SPEC_VIDEO              A2

#if defined(CORE_TEENSY)
#define FLASH_TRIGGER        12
IntervalTimer flashTimer;
#endif

String incomingStr,    datastring;
String    fileName,folderName;
  char fileNameType[100]= "test.txt";
  char folderNameType[12]= "";
uint16_t data [C12880_NUM_CHANNELS];
uint16_t dataseries [30][C12880_NUM_CHANNELS];
int timearray [30], numberOfSample;
uint16_t    peakvalue;
int pixeltarget=52;
elapsedMillis timeElapsed;
boolean flashstarted=false;
uint16_t    setintensity =12000;
File myFile;
const int chipSelect =    BUILTIN_SDCARD;

C12880_Class spec(SPEC_TRG,SPEC_ST,SPEC_CLK,SPEC_VIDEO);

/******************************************************************************/
void setup(){
    #if defined(CORE_TEENSY)
    pinMode(FLASH_TRIGGER,OUTPUT);
    digitalWrite(FLASH_TRIGGER,LOW);
    #endif
    Serial.begin(115200); // Baud Rate set to 115200

    if (!SD.begin(chipSelect)) {
        Serial.println("initialization failed!");
        return;
    }

    spec.begin();
    spec.set_integration_time(100);
}

```

```
void loop(){
//check if any input from serial port
    if (Serial.available () > 0){
    //send the input to process
    processIncomingByte (Serial.read ());
    }

}

// Subroutine to parse the input
void processData (String inputdata)
  {
    String inputString = inputdata;
    String timestamp;


  if (inputString.indexOf("IT")>-1)          //
    {
        String AcqTime= inputString.substring(inputString.indexOf(":")+1 );
        int integ_time=AcqTime.toInt();
        spec.set_integration_time(integ_time);
     }

    else if     (inputString.indexOf("Foldername")>-1)          //
    {
        folderName= inputString.substring(inputString.indexOf(":")+1 );
        folderName.toCharArray(folderNameType, sizeof(folderName));

     // see if the directory exists, create it if not.
        if( !SD.exists(folderNameType) )
        {
            SD.mkdir(folderNameType);
        }

      }
    else if     (inputString.indexOf("Filename")>-1)          //
     {
       fileName= inputString.substring(inputString.indexOf(":")+1 ) + ".txt";
       folderName += "/";
       fileName= folderName + fileName;
       fileName.toCharArray(fileNameType, sizeof(fileName)+4);
       myFile = SD.open(fileNameType, FILE_WRITE);
       myFile.print("      ");
    }

    else if     (inputString.indexOf("READ")>-1)          //
     {
        String sampleNum= inputString.substring(inputString.indexOf(":")+1 );
        int numberOfSample=sampleNum.toInt();
        readspecdata(numberOfSample);
      }

  inputString="";

}    // end of processData
```

```
void processIncomingByte (char c)
    {
    switch (c)
        {
        case '!':       // end of text
            // terminator reached! process inputLine here ...
            processData (incomingStr);
          delayMicroseconds(10);
            // reset for next time
            incomingStr="";
            break;

        default:
            // keep adding
            incomingStr += c;
            break;

        }    // end of switch
    } // end of processIncomingByte
void readspecdata(int numberOfSample)
{
  char readchar;
timeElapsed=0;
if (myFile)
    {
            for (int j = 0; j < 2000; j++)
            {
              spec.read_into(datastring, peakvalue);

              if (peakvalue>setintensity && !flashstarted)
                {
                      flashstarted=true;
                      j=2000-numberOfSample;
                }

              if (flashstarted){
                      myFile.print(timeElapsed,DEC);
                      myFile.print(":");
                      myFile.println(datastring + "!");
                }
            }
}

    // close the file:
myFile.close();


    // re-open the file for reading:
if (flashstarted)
    {
    myFile = SD.open(fileNameType);
    if (myFile)
        {
          datastring="";
        // read from the file until there's nothing else in it:
          readchar=myFile.read();
```

```
                readchar=myFile.read();
                  while (myFile.available())
                      {
                        readchar=myFile.read();
                        if (readchar != '!')
                        {
                            datastring+=readchar;
                        }
                        else
                        {
                            Serial.println(datastring.substring(0,768 + datastring.indexOf(":"))+">");
                            while(Serial.read() != '>')
                            {
                                    delayMicroseconds(10);
                            }
                           Serial.println(datastring.substring(769 + datastring.indexOf(":"),datastring.length()) + "!");
                            datastring="";
                            while(Serial.read() != '!')
                              {
                                  delayMicroseconds(10);
                              }
                        }
                      }

            // close the file:
            myFile.close();

        }
        else
            {
            // if the file didn't open, print an error:
            Serial.print("error opening ");
            Serial.println(fileNameType);
          }
            Serial.println ("*");
            flashstarted=false;
        }
}
/*********************************************************************************/
```
